# Coding-Complete Genome Sequence of an African Swine Fever Virus Strain Liv13/33 Isolate from Experimental Transmission between Pigs and Ornithodoros moubata Ticks

**DOI:** 10.1128/MRA.00185-20

**Published:** 2020-04-23

**Authors:** Amélie Chastagner, Rémi Pereira de Oliveira, Evelyne Hutet, Mireille Le Dimna, Frédéric Paboeuf, Pierrick Lucas, Yannick Blanchard, Linda Dixon, Laurence Vial, Marie-Frédérique Le Potier

**Affiliations:** a Swine Virology and Immunology Unit, ANSES Ploufragan-Plouzané-Niort Laboratory, Ploufragan, France; b UMR ASTRE, CIRAD, Montpellier, France; c UMR ASTRE, CIRAD, INRAE, University Montpellier, Montpellier, France; d SPF Pig Production and Experimentation Unit, ANSES Ploufragan-Plouzané-Niort Laboratory, Ploufragan, France; e Viral Genetic and Biosecurity Unit, ANSES Ploufragan-Plouzané-Niort Laboratory, Ploufragan, France; f OIE Reference Laboratory, The Pirbright Institute, Surrey, United Kingdom; DOE Joint Genome Institute

## Abstract

Here, we report the coding-complete genome sequence of African swine fever (ASF) virus strain Liv13/33, isolated from experimentally infected pigs and Ornithodoros moubata ticks. The 11 sequences that we obtained harbored no notable differences to each other, and all of them were closely related to the genome sequence of the Mkuzi 1979 strain of genotype I.

## ANNOUNCEMENT

African swine fever is a contagious and highly lethal disease of pigs and wild suids caused by the African swine fever virus (ASFV) (*Asfarviridae*, *Asfivirus*) and may involve soft ticks of the *Ornithodoros* genus as vectors and reservoirs of the virus (1).

The strain Liv13/33 was initially isolated in 1983 from a tick of the Ornithodoros moubata group in Livingstone (Zambia, Africa) (2, 3). This strain was previously sequenced on two genes (B646L/P72 and E182L/P54) and identified as belonging to the genotype I (4, 5). The coding-complete genome sequence of Liv13/33 presented in this report was obtained from samples of a study that experimentally tested the vector competence of *O. moubata sensu stricto* ticks on pigs as previously described (6). Three 7-week-old specific-pathogen-free (SPF) Large White pigs were inoculated by the intramuscular route with a 10^4^ of the 50% hemadsorbing dose (HAD_50_) of the ASFV Liv13/33 strain. Two hundred and sixty ASFV-free ticks were engorged on infected pigs on the first day of hyperthermia, when pig viremia ranged from 10^7.8^ to 10^8.1^ HAD_50_/ml. Five engorged ticks were frozen 3 months postinfection for sequencing analysis, and the others were fed on three new SPF pigs to assess their ability to transmit ASFV to naive pigs. These pigs displayed hyperthermia 2 days after being bitten by infected ticks.

Animal experiments performed at the air-filtered biosafety level 3 animal facilities at ANSES-Ploufragan were authorized by the French Ministry for Research (project no. 2017062615498464) and approved by the national ethics committee (authorization no. 11/07/17-3).

DNA was extracted using the High Pure PCR template preparation kit (Roche Life Science) from 400 μl of heparin blood samples collected from the six pigs during the viremia peak and from supernatants of crushed ticks prepared by filtration at 0.45 μm. All samples were sequenced at the ANSES Institute (Ploufragan, France) with Proton Ion Torrent technology (Thermo Fisher Scientific, Frederick, MD). Individual libraries were created for each of the 6 pig samples and each of the 5 infected ticks for a total of 11 libraries. The libraries for sequencing were prepared using the Ion Xpress plus fragment library kit and Ion Xpress barcode adapters 1-96 kit (Thermo Fisher Scientific). Magnetic beads from the Agencourt AMPure XP kit (Beckman Coulter, Villepinte, France) were used for DNA purification steps. The resulting reads were cleaned with Trimmomatic version 0.36 (options: ILLUMINACLIP: oligos.fasta: 2:30:5:1: true; LEADING: 3; TRAILING: 3; MAXINFO: 40:0.2; MINLEN: 36) and were first *de novo* assembled using the SPAdes version 3.10.0 (option: –careful -t 12 -m 50) and MIRA version 4.0.2 (option: IONTOR_SETTINGS -ASSEMBLY:mrpc = 100) programs. In parallel, reads were mapped on three reference ASFV genomes of genotype I (BA71 [GenBank accession no. KP055815], Mkuzi 1979 [AY261362], and Benin97/1 [AM712239]) using Burrows-Wheeler Aligner software version 0.7.15-r1140 (option: mem -M). For each library, contigs produced by the different methods were scaffolded to generate a single consensus sequence validated by an additional BWA alignment. *De novo* assemblers and alignment software could not deal with inverted terminal repeats (ITRs) present in the ASFV genomes (7); the obtained sequence was thus probably shorter than that in reality. A comparison of the 11 genomes obtained showed fewer than 7 nucleotide differences that were mainly in ITRs and up to 34 gaps located in mononucleotide repeats A or T (Table 1). The coding-complete genome sequence of Liv13/33 with the best coverage (72.09×) was isolated from tick OmLF2 (Table 1). This sequence of 188,277 bp (G+C content of 38.4%) harbored 228 open reading frames (ORFs) annotated with the help of Prokka (Galaxy version 1.13) based on the annotations of genomes available on the African Swine Fever Virus Database (http://asfvdb.popgenetics.net/), which proposed the most complete and homogeneous revised annotation (8).

**TABLE 1:** Description of Liv13/33 libraries generated in this study

Isolate	Host	Total no. of produced reads	Total no. of mapped reads	Mean coverage^*a*^ on reference sequence OmLF2^*b*^	No. of nucleotide differences/gaps compared to reference sequence OmLF2^*b*^	BioSample accession no.	SRA accession no.
6517IM	SPF pig inoculated by the intramuscular route	7,629,638	26,960	18.70	2/17	SAMN13195023	SRS6053714
6524IM	SPF pig inoculated by the intramuscular route	8,281,778	23,558	14.67	1/5	SAMN13195024	SRS6053715
6540IM	SPF pig inoculated by the intramuscular route	11,118,211	47,712	32.69	0/6	SAMN13195025	SRS6053707
OmLF1	Infected *O. moubata* tick	8,013,796	33,325	19.54	6/30	SAMN13191038	SRS6053706
OmLF2	Infected *O. moubata* tick	13,675,689	129,761	72.16	Reference	SAMN13191036	SRS6053705
OmLF3	Infected *O. moubata* tick	5,526,024	27,692	16.83	7/34	SAMN13191040	SRS6053708
OmLM1	Infected *O. moubata* tick	7,096,453	29,221	18.93	2/5	SAMN13191039	SRS6053709
OmLM2	Infected *O. moubata* tick	3,742,020	33,372	21.39	0/8	SAMN13191042	SRS6053710
6573T	SPF pig infected by ticks	12,708,781	26,414	17.14	1/10	SAMN13194022	SRS6053712
6594T	SPF pig infected by ticks	7,616,042	25,491	17.81	0/10	SAMN13195022	SRS6053713
893T	SPF pig infected by ticks	9,392,643	29,607	21.65	3/9	SAMN13191216	SRS6053711

a The mean coverage corresponds to the mean number of reads mapped on the sequence of reference by position.

b GenBank accession no. MN913970.

### Data availability.

The coding-complete genome sequence of isolate OmLF2 has been deposited in GenBank under the accession no. MN913970. Raw data from the 11 isolates for this project can be found in the GenBank SRA under accession no. PRJNA587575.
